# Application of ionomics to plant and soil in fields under long-term fertilizer trials

**DOI:** 10.1186/s40064-015-1562-x

**Published:** 2015-12-18

**Authors:** Toshihiro Watanabe, Masaru Urayama, Takuro Shinano, Ryosuke Okada, Mitsuru Osaki

**Affiliations:** Research Faculty of Agriculture, Hokkaido University, Kita-9, Nishi-9, Kitaku, Sapporo, 0608589 Japan; Agricultural Radiation Research Center, NARO Tohoku Agricultural Research Center, 50 Aza Harajyukuminami, Arai, Fukushima, 9602156 Japan

**Keywords:** Ionomics, Long-term fertilizer experiment field, Nitrogen, Phosphorus, Potassium

## Abstract

Ionomics is the study of elemental accumulation in living organisms using high-throughput elemental profiling. In the present study, we examined the ionomic responses to nutrient deficiency in maize grown in the field in long-term fertilizer trials. Furthermore, the available elements in the field soils were analyzed to investigate their changes under long-term fertilizer treatment and the ionomic relationships between plant and soil. Maize was cultivated in a field with the following five long-term fertilizer treatments: complete fertilization, fertilization without nitrogen, without phosphorus, without potassium, and no fertilization. Concentrations of 22 elements in leaves at an early flowering stage and in soils after harvest were determined. The fertilizer treatments changed the availabilities of many elements in soils. For example, available cesium was decreased by 39 % and increased by 126 % by fertilizations without nitrogen and potassium, respectively. Effects of treatments on the ionome in leaves were evaluated using the translocation ratio (the concentration in leaves relative to the available concentration in soils) for each element. Nitrogen deficiency specifically increased the uptake ability of molybdenum, which might induce the enhancement of nitrogen assimilation and/or endophytic nitrogen fixation in plant. Potassium deficiency drastically enhanced the uptake ability of various cationic elements. These elements might act as alternatives to K in osmoregulation and counterion of organic/inorganic anions. Two major groups of elements were detected by multivariate analyses of plant ionome. Elements in the same group may be linked more or less in uptake and/or translocation systems. No significant correlation between plant and soil was found in concentrations of many elements, even though various soil extraction methods were applied, implying that the interactions between the target and other elements in soil must be considered when analyzing mineral dynamics between plant and soil.

**Electronic supplementary material:**

The online version of this article (doi:10.1186/s40064-015-1562-x) contains supplementary material, which is available to authorized users.

## Background

Nitrogen (N), phosphorus (P), and potassium (K) are the three major fertilizer nutrients, and adequate fertilization is one of the most important factors in plant production (Mengel and Kirkby 2001). Fertilization may affect the availability of elements other than those applied to soils. For example, the application of ammonium sulfate may increase the availability of some heavy metals in soils by lowering soil pH (Murányi et al. 1994). The effects of fertilization on soils are more obvious in a long-term fertilizer experiment field. Malhi et al. (1998) continuously applied ammonium nitrate at different rates for 27 years and determined the chemical properties of the soils (thin Black Chernozem, Typic Boroll). They found that ammonium nitrate application reduced soil pH, increased KCl-extractable aluminum (Al) and diethylenetriaminepentaacetic acid (DTPA) extractable iron (Fe), and affected the concentration of DTPA-extractable manganese (Mn) and zinc (Zn). Likewise, Li et al. (2007) performed long-term fertilizer trials with the three major nutrients for 16 years and compared DTPA-extractable Fe, Mn, Zn, and copper (Cu) in soils among treatments. Thus, effects of long-term fertilization on element availability in soils have been reported mainly for specific (e.g., essential) elements. However, the soil contains various elements of the periodic table (Tyler and Olsson 2001).

Plants can absorb and transport nonessential as well as essential elements (Marschner 2012). The recent development of inductively coupled plasma mass spectroscopy (ICP-MS) has allowed simultaneous and rapid detection of macroelements and microelements in plants (Lahner et al. 2003). The term “ionome” has been defined to describe all metals, metalloids, and nonmetals present in an organism irrespective of their essentiality (Lahner et al. 2003). Till date, many ionomic studies have been conducted to examine the relationship between ionomes and genomes in model plants (Baxter et al. 2008; Chao et al. 2011; Sanchez et al. 2008). For example, Chen et al. (2009) performed an ionomic study using approximately 2000 EMS-treated *Lotus japonicus* mutants. The results suggested that the number of genes regulating essential elements was not larger than those controlling nonessential elements. In addition to these studies using model plants, ionomic studies on crops and wild plants have also been conducted to analyze their genetic or phylogenetic variation (Norton et al. 2010; Watanabe et al. 2007).

The changes in the rhizosphere environment also affect plant ionomes. Among them, the effects of nutrient status in rhizosphere on plant ionome have been studied well. Tomasi et al. (2014) reported that the recovery of Fe deficiency by the Fe-humic substance complexes was accompanied by the enhancement of other cationic nutrients accumulation and allocation into leaves. Likewise, Pii et al. (2015) examined the ionomic responses to Fe deficiency in different crop species under different growth conditions, hydroponic and soil conditions, and suggested that the ionome profile can be useful for the diagnosis of plant physiological and nutritional statuses. They also pointed out the importance of studying processes and mechanisms governing nutrient availability in soil conditions. In a field study, Hejcman et al. (2013) reported that long-term (56 years) fertilizer treatment (no fertilizer, chemical fertilizer, and chemical fertilizer with organic matter) changed the properties of available elements in soils but did not significantly affect the concentration of minerals in barley grains except the three major nutrients and Fe. Gunes et al. (2008; 2009) applied polarized energy dispersive x-ray fluorescence to examine the effect of sulfur application on the shoot ionome in maize and alfalfa grown in the field, and found that concentration of various essential and nonessential elements was changed by the sulfur application. White et al. (2012) analyzed the shoot ionome of herbage (21 plant species representing seven plant families) from six subplots of the Rothamsted Park Grass Experiment. Subplots had received contrasting fertilizer treatments [different application rates of N, P, K, sodium (Na), and magnesium (Mg)] since 1856. They observed that shoot ionomes were relatively sensitive to fertilizer treatment, whereas the phylogenetic variation in a subset of the shoot ionome [calcium (Ca), Zn, Mn, and Mg] was robust to fertilizer treatment.

Thus, nutrient deficiency may change mineral balances in plants. Also, fertilization can change mineral balances in soil indirectly affecting plant mineral accumulation. However, almost no experimental work has been reported on studying the effect of incomplete fertilization on both plant and soil ionome profiles comprehensively under field conditions. In the present study, we applied ionomics to examine the effect of long-term nonapplication of one or all the three major nutrients on mineral dynamics in plants and soil in which the plants were grown. Furthermore, we tried to identify hitherto unknown interactions between nutrient deficiency and the ionome, which would give new implications for plant responses to nutrient deficiency.

## Methods

### Cultivation

In 2009, maize (*Zea mays* L. cv. Yumeno-corn) was cultivated in the long-term fertilizer experimental field. This field was established in 1914, and five fertilizer treatments, complete fertilization (+NPK), without N (−N), without P (−P), without K (−K), and no fertilization (−NPK, started in 1921), have been continuously applied for 95 years. The cultivation history of the field is described in Additional file 1: Table S1. N, P, and K fertilizers were applied as ammonium sulfate, superphosphate, and potassium sulfate, respectively (100 kg N, P_2_O_5_, K_2_O ha^−1^), once before sowing. Each plot was 5.25 × 18.5 m in size, and the soil type was classified as a brown lowland soil (Haplic Fluvisols). General properties of the field soils were shown elsewhere (Cheng et al. 2013). Seeds of maize were sown on May 27 and plant shoots (leaf, stem, ear, and flower) were sampled on July 23 at the early flowering stage with 3 replications from at least 3 plants each. The row and hill spacing was 50 × 50 cm. After determination of fresh weight of shoots, leaves were separated from shoots, dried in an oven at 70 °C for 72 h, weighed, and ground with a vibrating sample mill (TI-100; CMT, Saitama, Japan) for mineral analysis. Soil samples were collected with 3 replications after harvest (September 18) from at least 5 points each (0–15 cm) and mixed in each replication. Although plant and soil samples were collected at different times because of various reasons in cultivation, seasonal variations in available concentration of minerals, except for N, P and K, in soils were negligible (data not shown). Soil samples were dried at room temperature and passed through a sieve with round holes of 2-mm diameter for mineral analysis.

### Analysis

Plant samples were digested in 2 mL of 61 % (w/v) HNO_3_ (EL grade; Kanto Chemical, Tokyo, Japan) at 110 °C in a DigiPREP apparatus (SCP Science, Canada) for approximately 2 h until the solution had almost disappeared. When the samples had cooled, 0.5 mL of H_2_O_2_ (semiconductor grade; Santoku Chemical, Tokyo, Japan) was added and the samples were heated at 110 °C for another 20 min. After digestion was complete, the tubes were cooled and filled to 10 mL with 2 % (w/v) HNO_3_ in Milli-Q water. The elements (lithium (Li), boron (B), Na, K, chromium (Cr), cobalt (Co), nickel (Ni), arsenic (As), molybdenum (Mo), cadmium (Cd), Mg, Al, P, Ca, Mn, Fe, Cu, Zn, strontium (Sr), barium (Ba), and Cs were analyzed using a ICP-MS (ELAN DRC-e; Perkin Elmer, Waltham, MA, USA). The air-dried soil samples were extracted with various extractants [0.1 and 1 M HCl (soil:HCl = 1:5, w/v), water (soil:Milli-Q water = 1:2.5, w/v), 0.25 M acetic acid (soil:acetic acid = 1:5, w/v), 1 M ammonium acetate (soil:ammonium acetate = 1:5, w/v)] for mineral analysis. Mineral concentration in the water and HCl extractants were determined directly by ICP-MS and inductively coupled plasma atomic emission spectrometry (ICP-AES), respectively. Mineral concentrations in the acetic acid and ammonium acetate extracts were determined using ICP-MS after digestion with HNO_3_ as described above. The soil samples were also digested with HNO_3_ and mineral concentrations were determined using ICP-MS_._ Available P was determined by the method of Truog (Truog 1930). Total N concentration in plant and soil samples was determined by micro-Kjeldahl digestion. Soil pH (soil:Milli-Q water = 1:2.5, w/v) was measured with a pH meter. For determination of inorganic N (NH_4_–N and NO_3_–N) concentration, soils were extracted with 2 M KCl, and NH_4_–N and NO_3_–N concentrations were determined with an autoanalyzer (AACS-3, Bran + Luebbe Norderstedt, Germany) following the manufacturer’s instructions.

### Statistics

All statistical analyses were performed with Microsoft Excel and Minitab 14 (Minitab Inc., State College, PA).

## Results

### Soil

The general chemical properties of soil determined by conventional methods for each treatment after harvest are shown in Table 1. The concentration of NO_3_–N was below the detection limit (10 µg g^−1^). The concentration of NH_4_-N was also low and no significant difference was found among the treatments. Total N concentration was significantly lower in the −N and −NPK treatments than in the +NPK treatment. Trends of changes in Truog P and exchangeable K among treatments were similar for total P and K; Truog P and total P were low in the −P and −NPK treatments and exchangeable K and total K were low in the −K treatment and −NPK treatments (Table 1). However, differences in concentration of total and exchangeable K were almost constant among all the fertilizer treatments (Table 1). Soil pH was slightly higher in the −N and −NPK treatments than in the other treatments.

**Table 1:** General chemical properties of soils after harvest

	NH_4_–N (µg g^−1^)	Total N (mg g^−1^)	Truog P (mg P_2_O_5_ kg^−1^)	Total P (mg g^−1^)	Exchangeable K (µg g^−1^)	Total K (µg g^−1^)	pH (H_2_O)
+NPK	53.8 ± 3.1 a	2.26 ± 0.04 ab	306.3 ± 10.4 a	1.86 ± 0.03 ab	425 ± 18 b	1143 ± 15 ab	5.34 ± 0.11 b
−N	54.9 ± 2.9 a	1.85 ± 0.06 c	261.1 ± 25.4 a	1.74 ± 0.06 b	747 ± 60 a	1554 ± 76 a	5.54 ± 0.05 ab
−P	57.5 ± 2.7 a	2.09 ± 0.07 bc	22.1 ± 3.1 b	0.83 ± 0.03 c	706 ± 23 a	1479 ± 25 a	5.28 ± 0.08 b
−K	56.7 ± 1.8 a	2.55 ± 0.06 a	317.3 ± 14.5 a	2.11 ± 0.03 a	114 ± 5 c	943 ± 6 c	5.31 ± 0.07 b
−NPK	53.4 ± 2.2 a	1.92 ± 0.05 c	26.1 ± 0.4 b	0.82 ± 0.03 c	125 ± 8 c	966 ± 31 bc	5.76 ± 0.05 a

+NPK, complete fertilization; −N, fertilization without N; −P, fertilization without P; −K, fertilization without K; −NPK, no fertilization. Values are means (n = 3) ± standard errors. Different letters indicate a significant difference (*P* < 0.05) using Tukey’s multiple-comparison test following a one-way ANOVA

**Fig. 1:**
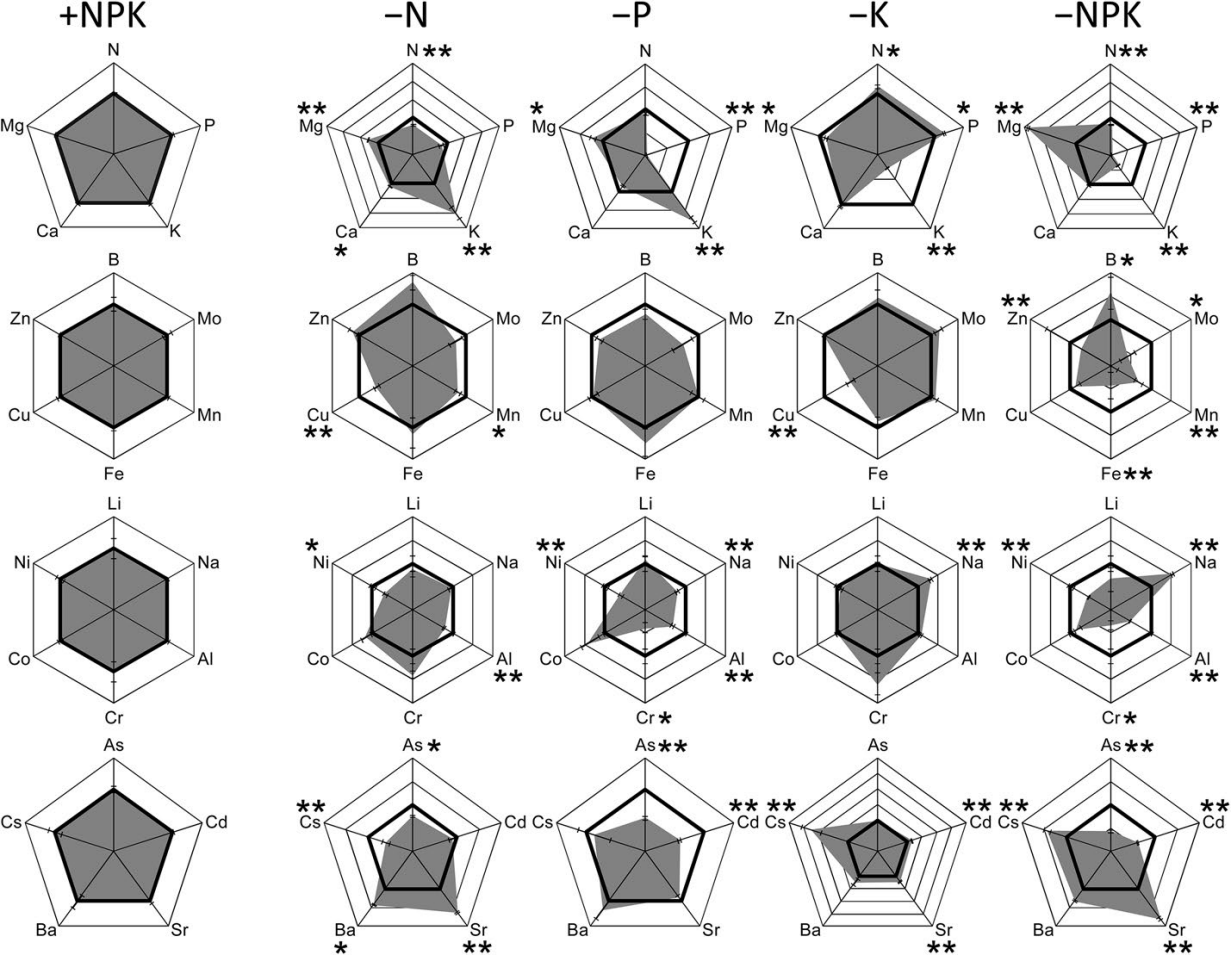
Radar plot representing the concentration of each element relative to the +NPK treatment. Concentrations of 0.1 M HCl-extractable elements, except those of N and Cs, are presented. For N and Cs, the water-extractable and total concentrations, respectively, are shown. The *bold line* indicates the relative concentration in the +NPK treatment (=1). +NPK, complete fertilization; −N, fertilization without N; −P, fertilization without P; −K, fertilization without K; −NPK, no fertilization. Values are means (*n* = 3), and bars indicate ± standard errors. *Asterisks* indicate statistically significant differences compared with +NPK (Student’s *t* test, *P < 0.05, **P < 0.01)

Mineral elements in soils were extracted with various extractants or digested with HNO_3_ to comprehensively estimate the available elements in soils. All results are summarized in Additional file 1: Table S2. Among them, concentrations of 0.1 M HCl extractable elements are shown in Fig. 1 as representative values except for N and Cs. Because the sensitivity of Cs determination is extremely low in ICP-AES, and Cl in the 0.1 M HCl extractant interferes with the detection of various elements in ICP-MS, water-extractable concentration determined using ICP-MS is shown for Cs. Total N is shown for the evaluation of soil N status, as significant treatment effects are described in Table 1. Concentration in each treatment is shown as a relative value to that in the +NPK treatment. Changes in concentration of P, K, Ca, and Mg extracted with 0.1 M HCl showed the same trend as determined by conventional methods (Table 1). Among the major nutrients, K concentration was higher in the −N and −P treatments, and N and P concentrations were higher in the −K treatment (Fig. 1). Mg concentration was higher in the −N, −P, and −NPK treatments. Among trace elements, Ni, Cr, As, and Cd concentrations were lower in the −P and −NPK treatments. Sr and Ba concentrations were higher in the −N treatment. Cs concentration was lower in −N and higher in the −K and −NPK treatments.

### Plant

**Fig. 2:**
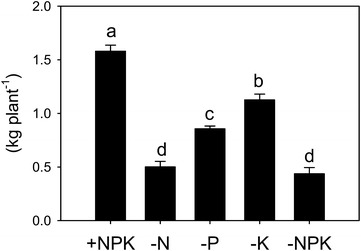
Fresh weights of maize shoots grown in a long-term fertilizer experiment field at an early flowering stage. +NPK, complete fertilization; −N, fertilization without N; −P, fertilization without P; −K, fertilization without K; −NPK, no fertilization. Values are means (*n* = 3), and bars indicate ± standard errors. Different letters indicate statistically significant difference (*P* < 0.05) using Tukey’s multiple comparison test following a one-way ANOVA

All nutrient-deficient treatments decreased the fresh weight of maize shoots at the early flowering stage compared with +NPK (Fig. 2). The fresh weights of maize shoots were in the following order: +NPK > −K > −P > −N, −NPK.

**Fig. 3:**
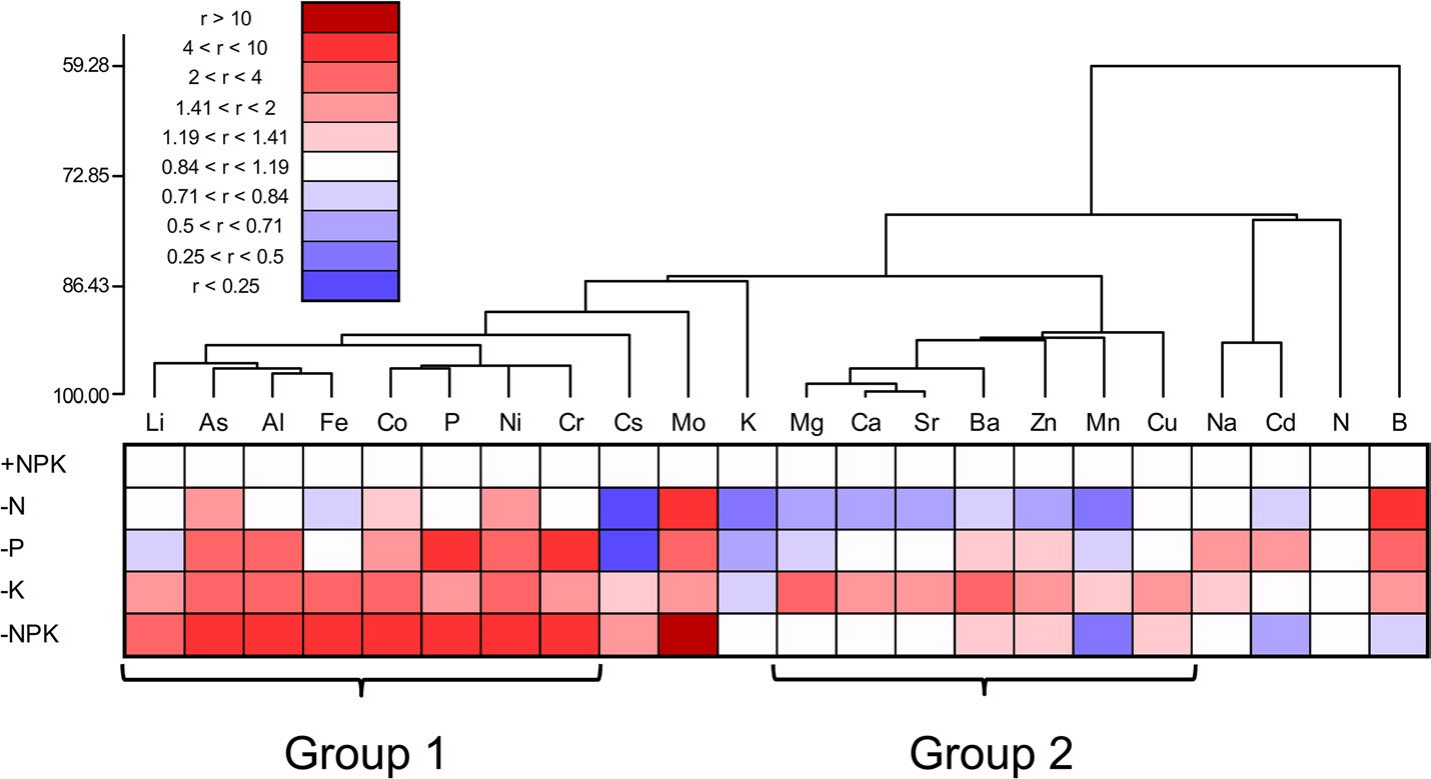
Heat-map analysis of changes in the translocation ratio of each element in maize grown under different nutrient conditions. +NPK, complete fertilization; −N, fertilization without N; −P, fertilization without P; −K, fertilization without K; −NPK, no fertilization. The translocation ratio for each element is the ratio of the concentration in leaves to the concentration in soil. For soil, concentrations of 0.1 M HCl-extractable elements, except those of N and Cs, are presented. For soil N and Cs concentrations, the water-extractable and total concentrations, respectively, are used. Hierarchical clustering was used to arrange the elements based on their translocation ratio. r: Relative translocation ratio with respect to the +NPK treatment

The mineral concentrations in maize leaves are shown in Additional file 1: Table S3. Because difference in availability of each element in soil can alter its accumulation in leaves, the effect of treatment was evaluated for each element as the translocation ratio, which is the ratio of concentration in leaves to the available concentration in soil. The data source for available element concentrations in soil is the same as for Fig. 1. Results are shown as a heatmap in Fig. 3. Hierarchical clustering was used to arrange the elements based on their relative concentrations. The translocation ratios of Ni, Al, As, Cr, P, Mo, and B showed increases in the −P treatment (Fig. 3; relative element concentration >1.41). Translocations ratio in most elements increased in the −K treatment. Translocation ratios of some alkaline and alkaline-earth metals, especially Cs, tended to decrease in the −N treatment. Marked increases in translocation ratio were observed for Mo in the −N and −NPK treatments and for B in the −N treatment. The translocation ratios of Ca, Sr, and Mg changed similarly in response to the fertilizer treatments.

**Fig. 4:**
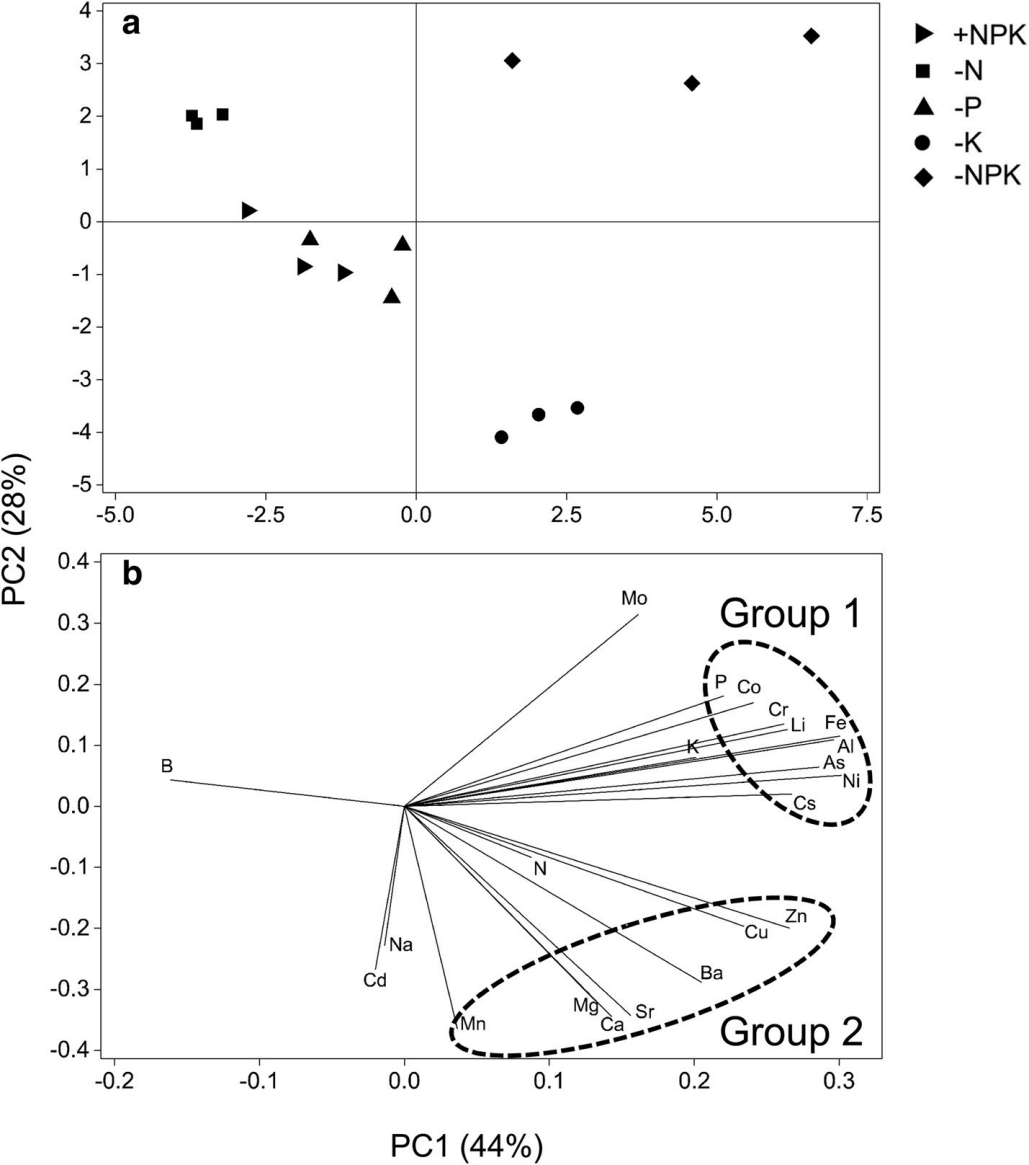
Principal component analysis (PCA) of elements in leaves of maize. +NPK, complete fertilization; −N, fertilization without N; −P, fertilization without P; −K, fertilization without K; −NPK, no fertilization. For the PCA analysis, values of the translocation ratio for each element were used. **a** Scores on the first two components (PC1 and PC2). **b** Loading plot for PC1 and PC2

The profile of elements (ionome) in plants was compared among treatments with principal component analysis (PCA) using the translocation ratio for each element as a variable. PCA scores are presented on the basis of combinations of PC1 and PC2 (Fig. 4). PC1 and PC2 accounted for 44 and 28 % of the total variance, respectively. The score plot of PCA showed major differences in leaf element composition between the K-deficient treatments (−K, −NPK) and the other treatments (Fig. 4a).

Correlation coefficients between leaf and soil concentrations were calculated for each element and each soil analysis (Table 2). No significant correlation was observed for most elements, but high correlation coefficients were found in several cases (N in Total N, Fe in ammonium acetate extraction, Mn in 0.1 M HCl extraction, Cd in water extraction, and Cs in water, acetic acid, and ammonium acetate extractions).

**Table 2:** Correlation coefficients between concentrations of elements in soil and maize leaves

Soil analysis procedure	N	P	K	Ca	Mg	Fe	Mn	Zn	Cu	Mo	B	Li	Na	Al	Cr	Co	Ni	As	Cd	Sr	Ba	Cs
0.1 M HCl extraction	–	0.828^†^	0.834^†^	0.329	0.062	−0.612	0.910*	−0.148	0.643	−0.693	0.266	−0.653	0.566	−0.134	−0.313	−0.261	−0.419	0.261	0.773	0.247	0.061	–
1 M HCl extraction	–	0.813^†^	0.826^†^	0.307	0.388	−0.277	0.731	0.177	0.179	−0.328	0.454	0.529	0.687	−0.101	−0.542	−0.198	−0.475	0.319	0.673	0.276	0.392	–
Water extraction	–	0.708	0.823^†^	0.594	−0.224	−0.262	0.861^†^	0.471	−0.754	–	0.455	−0.408	0.490	−0.038	−0.780	−0.165	−0.682	0.149	0.927*	0.123	0.124	0.914*
Acetic acid extraction	–	0.734	0.828^†^	0.335	0.068	0.010	0.694	−0.040	−0.355	–	0.083	−0.598	0.530	−0.073	−0.296	0.181	−0.591	0.074	0.863^†^	0.026	0.026	0.985**
Ammonium acetate extraction	–	0.662	0.866^†^	0.118	0.020	0.897*	0.663	0.018	0.584	–	–	−0.428	0.572	0.122	0.122	0.248	−0.366	0.237	0.82^†^	0.377	0.133	0.976**
HNO_3_ digestion	–	0.802	0.785	0.348	−0.211	−0.764	0.628	0.818^†^	0.377	−0.541	−0.278	0.258	0.719	−0.145	−0.027	0.563	0.020	−0.506	0.721	0.276	0.637	0.710
Conventional method^a^	0.928*	0.837^†^	–	–	–	–	–	–	–	–	–	–	–	–	–	–	–	–	–	–	–	–

^a^Total N for N, Truog-P for P

^†^ *P* < 0.10, * *P* < 0.05, ** *P* < 0.01

The ratios of some essential elements to their homologous nonessential elements in leaves and soils (0.1 M HCl extraction) were individually calculated using the average concentration data of all treatments to assess the relative specificity of uptake of the essential element. There was no marked difference in Ca/Sr ratio between leaves and soils, whereas for K/Na, Ca/Ba, Mg/Sr, Mg/Ba, P/As, and Zn/Cd the ratio was much higher in leaves than in soils (Table 3).

**Table 3:** Ratio of some essential elements to their homologous nonessential elements in leaves and soils (0.1 M HCl extraction)

	K/Na	Ca/Sr	Ca/Ba	Mg/Sr	Mg/Ba	P/As	Zn/Cd
Soil	5	180	79	14	6	50	33
Leaf	93	318	1602	201	1016	13,059	204

## Discussion

Although many studies have reported changes of available elements in soils under long-term fertilizer application, the number of elements investigated has been relatively limited (see also the "Introduction" section). For example, concentrations of 11 elements (K, Ca, Mg, Na, Al, Ni, Mn, Cu, Zn, Cd, and Pb) were determined in field soils from the >100-year-old experiment in Geescroft and Broadbalk Wildernesses and Park Grass at Rothamsted Experimental Station, the best-known long-term fertilizer experiment fields in the world. In the same fields, they also determined other five elements including essential elements (Blake et al. 1999; Blake and Goulding 2002). In the present study, we comprehensively investigated the effects of long-term application of chemical fertilizer on the availability of 22 elements in soils. Moreover, various analyses, including HNO_3_ digestion, were performed to estimate the availability of each element in soils. Concentrations obtained by 0.1 M HCl extraction, which is often used to estimate available heavy metals in soils (DePaula and Mozeto 2001), were highly correlated with those obtained by ammonium acetate extraction for K, Ca, Mg, and Truog P (Additional file 1: Table S4). Therefore, concentrations of 0.1 M HCl-extractable elements, except those of N and Cs, are accordingly presented in Fig. 1 as representative values. For N and Cs, the water-extractable and total concentrations, respectively, are shown instead of those of 0.1 M HCl. For the three major nutrients, the K concentration in soil was higher in the −N and −P treatments, and N and P concentrations were higher in the −K treatment than in the +NPK treatment (Fig. 1). These results can be explained by assuming that the excess fertilizer applied to the fields has accumulated every year because of the poor growth of crops grown under a nutrient-deficient treatment (Fig. 2).

It is striking that the concentrations of some heavy metals (Ni, Cr, As, and Cd) were low in soils with the −P and −NPK treatments (Fig. 1), given that fertilization with P (as superphosphate) is expected to increase their availability in soils and/or to supply them to soils. Phosphate rock often contains heavy metals (Sabiha-Javied et al. 2009).

In the −K treatment, water-extractable as well as acetic acid- and ammonium sulfate-extractable Cs concentrations in soils were markedly higher than those in the control (+NPK) treatment (Fig. 1, Additional file 1: Table S2). Cs, which like K is an alkaline metal, adsorbed to soil may be displaced by K and leached below the rhizosphere when the K fertilizer is applied. In contrast, Cs concentrations were substantially lower in the −N treatment than in the control treatment, indicating that N (NH_4_) application increases available Cs in soils. Because ${\text{NH}}_{4}^{ + }$ and K^+^ are both monovalent cations and have a similar ionic radius (Jung et al. 2005), ${\text{NH}}_{4}^{ + }$ would be expected to displace Cs adsorbed to soil as well as K^+^. In fact, ammonium acetate extracted the soil Cs more efficiently than water (Additional file 1: Table S2). The reason why the application of NH_4_, but not of K, increases Cs availability in soils remains unknown.

As described above, long-term application or nonapplication of the three major nutrients substantially affects the profile of available elements in soil other than those applied as nutrient element(s) to soil. To understand the ionomic responses to deficiency of the three major nutrients physiologically, the effects of treatments on the accumulation of elements in leaves were evaluated using the translocation ratio (the concentration in leaves relative to the available concentration in soils) for each element. The results of hierarchical clustering corresponded closely to those of the loading plot of Principal component analysis (PCA) (Figs. 3, 4b). Two major groups of elements were detected: group 1, including Li, Fe, Ni, Al, Co, As, Cr, and P and group 2, including Mg, Ca, Sr, Ba, Cu, and Zn. Elements in the same group may partly share the same absorption and/or transport system(s) in plants. In group 1, P and As are the homologous elements in the periodic table. The dominant form of inorganic As in aerobic soils is arsenate, a strong competitive physiological analog of phosphate in plants, and plant roots absorb arsenate via a phosphate transporter (Zhao et al. 2009). In the Al–Fe relationship, the Al uptake competitively inhibits Fe uptake to avoid Fe toxicity in some Al accumulator species (Hajiboland et al. 2013; Watanabe et al. 2006), suggesting an interaction between Al and Fe in their transport systems in plants. In group 2, all the alkaline earth metals were included, and Ca and Sr in particular showed closely similar behavior (Fig. 3). Drouet and Herbauts (2008) found that Sr and Ca behaved similarly in all soil and tree compartments of two different forest ecosystems. A highly significant correlation was found between Ca and Sr concentrations in leaves of 138 plant families growing under different environmental conditions (Watanabe et al. 2007). With respect to the ratio of essential elements to their homologous nonessential elements in leaves and soils (by 0.1 M HCl extraction) individually, there was little difference between plant and soil Ca/Sr ratios, whereas the ratios in leaves were much higher for the other combinations (K/Na, Ca/Ba, Mg/Sr, Mg/Ba, P/As, and Zn/Cd; Table 3). This result suggests that Sr is taken up by the plant with selectivity similar to that of Ca, which is in contrast to higher specificity in the uptake of the other essential elements. White (2001) suggested that no competition or interactions between Ca, Ba, and Sr occur during transport to the shoot, possibly because of substantial apoplastic transport. In addition to these predictable interactions between elements, these element groupings may indicate novel interaction mechanisms between (among) different elements in plants.

N deficiency decreased the uptake ability of many elements, whereas the Mo uptake ability was increased (Fig. 3). Mo is bound to proteins and comprises the Mo cofactor (MoCo) in plants. One of the major Mo enzymes is nitrate reductase (Mendel 2011). Fan et al. (2007) reported that rice cultivars showed higher nitrate reductase activity under N deficiency conditions. Because the rapid conversion of nitrate to ammonium can contribute to rapid protein synthesis, plants may enhance Mo uptake to maintain a higher nitrate reductase activity under N deficiency conditions. In fact, Yu et al. (2010) reported that the application of Mo to wheat enhanced nitrate reductase activity and reduced nitrate concentration. Moreover, Mo is a constituent of the FeMo cofactor in bacterial nitrogenase (Mendel 2011). Because endophytic N fixation occurs in maize (James 2000), it is possible that N deficiency increases the N fixation activity in endophytic bacteria, resulting in Mo acquisition by the host plant.

P deficiency increased the P uptake ability in maize (Fig. 3). Plants have various mechanisms for acquiring P from the soil, and these mechanisms are stimulated by P deficiency (Raghothama 1999). P deficiency also increased the uptake ability of various elements in group 1 (Fig. 3). It is not surprising that As uptake is enhanced under P deficiency conditions, given that arsenate is an analog of phosphate, as indicated above. The increased uptake ability of other elements under P deficiency may be related to increased availability of these elements in rhizosphere soils by the P-deficiency-induced release of organic acids and/or H^+^ from roots (Liu et al. 2004; Gaume et al. 2001). Moreover, enhanced mycorrhizal colonization under P deficiency can increase uptake of some metal elements as well as P (Liu et al. 2000).

K deficiency drastically enhanced the uptake ability of various elements (Fig. 3). These changes led to the different distributions of the −K and −NPK treatments in the score plot of PCA (Fig. 4a). Similar results were reported in hydroponically grown tomatoes (Pujos and Morard 1997). The major roles of K in plants are osmoregulation and acting as a counterion of organic and inorganic anions (Marschner 2012). Under K deficiency conditions, plants may efficiently absorb other cations as alternatives to K. Among these cations, the effect of K deficiency on Cs uptake has been well studied. While Cs toxicity in plant is not a general problem, the pollution by radioactive Cs from the nuclear accident can also be a risk for human health in crop production. Enhanced Cs uptake under K deficiency can be attributed to two factors. The first is competitive inhibition of Cs uptake by K in plants (White and Broadley 2000), and the second is the involvement of membrane transporters. In *Arabidopsis*, K deficiency induces the expression of *AtHAK5*, encoding a high-affinity K^+^ transporter (Gierth et al. 2005), which is localized on the plasma membrane of root cells and transports Cs efficiently (Qi et al. 2008).

Although elements in soils were extracted by various extractants in the present study, no significant correlation was observed between plant and soil in concentrations of many elements (Table 2). Therefore, a single extraction method for a single element, whether or not conventional, cannot in most cases estimate plant its available concentration in soil. In order to estimate the plant available element in soil more accurately, the multifactorial data analysis of the interactions between the target and other elements in soil, obtained from various extraction methods, must be applied when analyzing element dynamics between plant and soil.

## Conclusions

Whereas ionomics has been used to date to examine the effects of genetic differences or gene modification on plant mineral uptake, the results of the present study show that it can also be useful for characterizing plant responses to changes in the soil environment. However, only a single unrepeated field experiment was conducted, and only one plant species was used in this study. Further field experiments are required using different plant species to confirm the repeatability and to examine the difference in response among different plant species. Another important finding of this study is that ionomics can also be applied to soil to capture the complex dynamics of elements in soil. More detailed elemental interaction between plant and soil may be revealed by increasing the number of sampling times and/or analyzing rhizosphere soils.

## Additional file

10.1186/s40064-015-1562-x In the supplemental material section cultivation history of the field used in this study, concentration data of each element in maize leaf and soil, and correlation coefficient between concentrations in soil determined by two different extraction methods for each element are presented.
