# Supplementary material for: Application of ionomics to plant and soil in fields under long-term fertilizer trials
Source: Springerplus. 2015 Dec 18;4:781. doi: 10.1186/s40064-015-1562-x (PMC4684559; doi:10.1186/s40064-015-1562-x)
Supplement: Supplementary file 1 — 10.1186/s40064-015-1562-x In the supplemental material section cultivation history of the field used in this study, concentration data of each element in maize leaf and soil, and correlation coefficient between concentrations in soil determined by two different extraction methods for each element are presented. [file 40064_2015_1562_MOESM1_ESM.pdf]

Table S1 Cultivation histry of the field used in this study.

1914-1935	Flax, Oat, Soybean, Sugar beet
1936-1965	No cultivation data
1966-1972	Alfalfa, Ladino Clover, Orchardgrass, Timothy
1973-1974	Maize
1975	Maize, Soybean, Tomato
1976-1977	No cultivation data
1978-1981	Maize, Potato, Soybean, Sugar beet
1982	Soybean
1983	Maize
1984	No planting
1985	Maize
1986	Potato
1987	Potato, Maize, Sweet potato
1988	Potato, Maize, Wheat, Soybean
1989	Potato, Sweet potato
1990	Sweet potato
1991	No cultivation data
1992	Maize
1993	Maize, Soybean, Wheat
1994	No cultivation data
1995	Amaranthus, Jerusalem artichoke, Maize, Soybean, Sunflower, Sweet Potato, Wheat
1996	No cultivation data
1997-1998	Oat
1999	Adzuki bean
2000	Oat
2001	Amaranthus, Common reed, Kenaf, Poplar, Zucchini
2002	White lupin
2003-2004	Maize, Sugar beat, Wheat, White lupin
2005	Maize, Soybean
2006	Soybean
2007	Sunflower
2008	Soybean
2009	Maize

Table S2 Concentration of each element in soils with different fetrtilizer treatment, determined by different method.

0.1 M HCl extract

	P (μg g^{-1})	K (μg g^{-1})	Ca (μg g^{-1})	Mg (μg g^{-1})	Fe (μg g^{-1})	Mn (μg g^{-1})	Zn (μg g^{-1})	Cu (μg g^{-1})	Mo (μg g^{-1})	B (μg g^{-1})	Li (μg g^{-1})	Na (μg g^{-1})	Al (μg g^{-1})	Cr (μg g^{-1})	Co (μg g^{-1})	Ni (μg g^{-1})	As (μg g^{-1})	Cd (μg g^{-1})	Sr (μg g^{-1})	Ba (μg g^{-1})
+NPK	273 ± 5	175 ± 3	3243 ± 31	183 ± 3	15.6 ± 0.3	41.5 ± 0.6	13.9 ± 0.2	1.037 ± 0.030	0.210 ± 0.009	0.342 ± 0.022	0.061 ± 0.006	36.0 ± 0.1	1357 ± 20	0.084 ± 0.008	0.175 ± 0.004	1.213 ± 0.047	4.38 ± 0.13	0.465 ± 0.003	13.8 ± 0.3	33.4 ± 2.5
-N	223 ± 10	348 ± 19	3470 ± 16	238 ± 1	16.8 ± 2.2	34.5 ± 1.1	15.4 ± 0.6	0.698 ± 0.030	0.168 ± 0.015	0.463 ± 0.024	0.053 ± 0.005	33.6 ± 0.9	1048 ± 12	0.116 ± 0.004	0.199 ± 0.015	0.820 ± 0.032	3.34 ± 0.05	0.412 ± 0.019	22.5 ± 0.1	47.4 ± 0.3
-P	5 ± 0	307 ± 7	2727 ± 35	223 ± 6	19.2 ± 2.0	40.4 ± 2.8	11.9 ± 0.4	1.005 ± 0.048	0.146 ± 0.022	0.287 ± 0.015	0.065 ± 0.003	28.0 ± 0.8	900 ± 25	0.032 ± 0.005	0.242 ± 0.022	0.751 ± 0.030	2.36 ± 0.06	0.268 ± 0.007	12.6 ± 0.3	38.8 ± 3.6
-K	313 ± 2	46 ± 1	3416 ± 23	164 ± 2	13.6 ± 0.5	43.8 ± 1.1	13.9 ± 0.1	0.528 ± 0.044	0.237 ± 0.008	0.377 ± 0.025	0.060 ± 0.002	47.0 ± 0.6	1444 ± 14	0.133 ± 0.012	0.173 ± 0.006	1.108 ± 0.023	4.29 ± 0.01	0.529 ± 0.004	16.6 ± 0.1	40.7 ± 2.3
-NPK	4 ± 0	57 ± 1	3345 ± 5	441 ± 2	6.4 ± 0.3	28.2 ± 0.5	10.0 ± 0.2	0.896 ± 0.014	0.086 ± 0.021	0.540 ± 0.014	0.040 ± 0.009	54.8 ± 0.7	632 ± 6	0.027 ± 0.005	0.144 ± 0.008	0.675 ± 0.006	1.90 ± 0.06	0.280 ± 0.004	24.7 ± 0.3	43.9 ± 0.3

1.0 M HCl extract

	P (μg g^{-1})	K (μg g^{-1})	Ca (μg g^{-1})	Mg (μg g^{-1})	Fe (μg g^{-1})	Mn (μg g^{-1})	Zn (μg g^{-1})	Cu (μg g^{-1})	Mo (μg g^{-1})	B (μg g^{-1})	Li (μg g^{-1})	Na (μg g^{-1})	Al (μg g^{-1})	Cr (μg g^{-1})	Co (μg g^{-1})	Ni (μg g^{-1})	As (μg g^{-1})	Cd (μg g^{-1})	Sr (μg g^{-1})	Ba (μg g^{-1})
+NPK	1084 ± 22	149 ± 5	5461 ± 185	509 ± 15	3212 ± 99	207 ± 4	24.8 ± 0.6	10.7 ± 0.4	0.755 ± 0.022	6.75 ± 0.19	0.191 ± 0.007	36.3 ± 0.2	3348 ± 24	1.193 ± 0.012	1.716 ± 0.033	2.700 ± 0.050	20.4 ± 0.4	0.773 ± 0.016	16.3 ± 0.1	69.7 ± 0.7
-N	1004 ± 24	293 ± 15	6872 ± 101	515 ± 6	3534 ± 86	174 ± 4	25.8 ± 0.7	9.7 ± 0.2	0.672 ± 0.018	6.92 ± 0.05	0.182 ± 0.003	33.3 ± 1.1	3084 ± 17	1.428 ± 0.050	1.618 ± 0.026	1.834 ± 0.085	19.0 ± 0.1	0.716 ± 0.022	26.7 ± 0.1	75.4 ± 0.6
-P	93 ± 3	250 ± 9	4056 ± 148	466 ± 8	2253 ± 54	135 ± 5	21.5 ± 0.5	8.5 ± 0.2	0.460 ± 0.004	4.37 ± 0.13	0.147 ± 0.011	27.0 ± 0.6	2768 ± 10	0.354 ± 0.014	1.118 ± 0.038	1.516 ± 0.033	11.9 ± 0.1	0.429 ± 0.005	15.4 ± 0.2	66.3 ± 1.5
-K	1271 ± 11	45 ± 0	6519 ± 127	536 ± 2	3290 ± 2	222 ± 1	26.6 ± 0.2	11.3 ± 0.1	0.769 ± 0.026	6.30 ± 0.02	0.242 ± 0.001	53.0 ± 3.4	3477 ± 10	1.213 ± 0.014	1.922 ± 0.018	2.387 ± 0.008	20.8 ± 0.0	0.826 ± 0.008	20.4 ± 0.1	77.9 ± 0.2
-NPK	111 ± 4	53 ± 1	6241 ± 28	797 ± 2	2426 ± 14	144 ± 3	17.9 ± 0.3	12.0 ± 0.1	0.508 ± 0.013	4.86 ± 0.03	0.184 ± 0.001	54.3 ± 1.1	2641 ± 3	0.465 ± 0.007	1.260 ± 0.023	1.418 ± 0.015	12.8 ± 0.0	0.479 ± 0.008	29.3 ± 0.4	68.0 ± 0.4

Water extract

	P (μg g^{-1})	K (μg g^{-1})	Ca (μg g^{-1})	Mg (μg g^{-1})	Fe (ng g^{-1})	Mn (ng g^{-1})	Zn (ng g^{-1})	Cu (ng g^{-1})	Mo (ng g^{-1})	B (ng g^{-1})	Li (ng g^{-1})	Na (ng g^{-1})	Al (ng g^{-1})	Cr (ng g^{-1})	Co (ng g^{-1})	Ni (ng g^{-1})	As (ng g^{-1})	Cd (ng g^{-1})	Sr (ng g^{-1})	Ba (ng g^{-1})	Cs (ng g^{-1})
+NPK	3.29 ± 0.33	19.2 ± 1.5	72.1 ± 7.8	5.58 ± 0.56	536 ± 96	465 ± 80	73.1 ± 10.5	21.2 ± 2.4	ND	179 ± 26	8.65 ± 1.25	14.0 ± 0.2	1.54 ± 0.63	8.34 ± 0.69	1.64 ± 0.15	14.3 ± 0.9	13.9 ± 1.4	0.570 ± 0.081	219 ± 25	86.0 ± 7.2	3.05 ± 0.19
-N	4.67 ± 0.81	35.7 ± 7.5	52.4 ± 7.1	4.88 ± 0.59	599 ± 58	153 ± 18	30.5 ± 2.3	20.2 ± 0.7	ND	229 ± 35	3.75 ± 0.37	12.4 ± 0.9	0.73 ± 0.43	7.18 ± 0.43	1.03 ± 0.10	11.0 ± 0.5	23.8 ± 2.6	0.200 ± 0.008	221 ± 32	56.7 ± 7.1	1.86 ± 0.14
-P	0.02 ± 0.02	32.0 ± 3.7	39.8 ± 12.5	4.53 ± 1.40	148 ± 12	531 ± 236	57.9 ± 20.0	6.6 ± 0.5	ND	137 ± 8	5.93 ± 1.78	9.0 ± 1.4	0.42 ± 0.21	3.17 ± 0.21	2.18 ± 0.84	4.3 ± 0.7	1.8 ± 0.1	0.318 ± 0.130	156 ± 48	83.2 ± 12.0	2.63 ± 0.48
-K	4.50 ± 0.48	8.0 ± 1.9	73.4 ± 9.5	4.49 ± 0.69	590 ± 283	493 ± 109	61.7 ± 11.8	16.0 ± 1.6	0.305 ± 0.305	157 ± 28	7.55 ± 1.01	16.5 ± 1.2	1.75 ± 3.94	5.41 ± 0.66	1.84 ± 0.42	11.8 ± 3.3	16.0 ± 1.0	0.525 ± 0.083	236 ± 31	83.6 ± 8.0	6.87 ± 0.58
-NPK	0.22 ± 0.03	4.8 ± 0.2	27.8 ± 0.6	5.49 ± 0.08	73 ± 3	86 ± 3	9.2 ± 1.6	6.8 ± 0.1	ND	227 ± 29	0.92 ± 0.04	15.7 ± 0.3	0.09 ± 0.03	3.45 ± 0.22	0.35 ± 0.01	1.7 ± 0.1	1.9 ± 0.0	0.062 ± 0.016	149 ± 3	32.6 ± 0.3	4.39 ± 0.13

HNO_3 digestion

	P (μg g^{-1})	K (μg g^{-1})	Ca (μg g^{-1})	Mg (μg g^{-1})	Fe (μg g^{-1})	Mn (μg g^{-1})	Zn (μg g^{-1})	Cu (μg g^{-1})	Mo (μg g^{-1})	B (μg g^{-1})	Li (μg g^{-1})	Na (μg g^{-1})	Al (μg g^{-1})	Cr (μg g^{-1})	Co (μg g^{-1})	Ni (μg g^{-1})	As (μg g^{-1})	Cd (μg g^{-1})	Sr (μg g^{-1})	Ba (μg g^{-1})	Cs (μg g^{-1})
+NPK	1979 ± 39	1143 ± 15	4917 ± 266	4310 ± 70	27419 ± 401	1325 ± 18	113 ± 1	39.1 ± 0.2	1.973 ± 0.594	5.398 ± 1.815	11.6 ± 0.2	240 ± 18	21343 ± 423	17.1 ± 0.1	14.7 ± 0.2	212 ± 2	49.4 ± 1.2	0.599 ± 0.027	25.6 ± 0.8	106 ± 1	7.08 ± 0.04
-N	1769 ± 109	1554 ± 76	5770 ± 214	4430 ± 38	28428 ± 396	1124 ± 52	112 ± 4	34.6 ± 0.8	0.784 ± 0.024	1.757 ± 0.250	12.8 ± 0.1	274 ± 18	22993 ± 541	16.6 ± 0.4	14.8 ± 0.6	223 ± 1	57.2 ± 1.9	0.514 ± 0.032	38.5 ± 0.7	108 ± 3	6.76 ± 0.24
-P	785 ± 24	1479 ± 25	4465 ± 366	4303 ± 127	28021 ± 342	1166 ± 31	121 ± 7	33.4 ± 0.2	0.636 ± 0.049	0.798 ± 0.119	12.3 ± 0.6	287 ± 44	22601 ± 1271	14.9 ± 0.4	14.6 ± 0.1	214 ± 6	49.3 ± 0.9	0.353 ± 0.011	26.6 ± 0.5	102 ± 2	7.25 ± 0.15
-K	2093 ± 21	943 ± 6	5859 ± 103	4182 ± 129	27132 ± 373	1293 ± 35	121 ± 3	38.9 ± 1.0	1.243 ± 0.384	2.664 ± 1.453	12.2 ± 0.2	340 ± 24	22738 ± 350	18.2 ± 0.6	14.9 ± 0.5	211 ± 5	45.9 ± 0.4	0.680 ± 0.029	31.3 ± 0.4	115 ± 3	7.40 ± 0.07
-NPK	757 ± 6	966 ± 31	6039 ± 39	4617 ± 43	27305 ± 103	1259 ± 34	115 ± 2	41.1 ± 0.2	0.827 ± 0.047	0.966 ± 0.086	12.8 ± 0.2	381 ± 18	20974 ± 370	16.0 ± 0.4	15.8 ± 0.3	221 ± 3	46.6 ± 0.6	0.428 ± 0.011	42.8 ± 0.5	106 ± 1	7.28 ± 0.10

Acetic acid extract

	P (μg g^{-1})	K (μg g^{-1})	Ca (μg g^{-1})	Mg (μg g^{-1})	Fe (μg g^{-1})	Mn (μg g^{-1})	Zn (μg g^{-1})	Cu (ng g^{-1})	Mo (ng g^{-1})	B (ng g^{-1})	Li (ng g^{-1})	Na (μg g^{-1})	Al (μg g^{-1})	Cr (ng g^{-1})	Co (ng g^{-1})	Ni (ng g^{-1})	As (ng g^{-1})	Cd (ng g^{-1})	Sr (μg g^{-1})	Ba (μg g^{-1})	Cs (ng g^{-1})
+NPK	23.8 ± 1.7	91 ± 5	1267 ± 44	76 ± 1	2.51 ± 0.14	9.73 ± 1.21	2.59 ± 0.18	43.9 ± 4.2	1.50 ± 1.50	423.2 ± 48.3	50.3 ± 3.3	27.7 ± 0.8	57.4 ± 2.7	26.9 ± 1.8	38.0 ± 4.7	221.2 ± 14.2	64.8 ± 2.7	31.9 ± 0.9	4.31 ± 0.06	3.46 ± 0.47	11.1 ± 1.4
-N	27.0 ± 2.4	187 ± 17	1513 ± 14	102 ± 1	3.06 ± 0.42	5.63 ± 0.22	2.66 ± 0.40	29.5 ± 1.4	ND	439.7 ± 32.1	46.1 ± 1.8	26.9 ± 1.4	36.8 ± 1.4	36.2 ± 11.1	29.6 ± 1.3	140.1 ± 6.4	97.2 ± 4.5	26.5 ± 0.6	7.23 ± 0.16	5.69 ± 0.14	6.6 ± 0.7
-P	1.7 ± 0.1	165 ± 7	1055 ± 40	96 ± 4	2.07 ± 0.03	14.12 ± 2.54	2.51 ± 0.17	25.4 ± 5.7	ND	297.3 ± 26.8	49.2 ± 5.4	21.6 ± 1.0	26.5 ± 2.0	23.2 ± 3.1	74.4 ± 14.9	158.6 ± 11.1	19.9 ± 2.2	23.6 ± 1.1	4.32 ± 0.14	5.33 ± 1.03	9.7 ± 0.7
-K	28.0 ± 1.2	22 ± 1	1488 ± 31	67 ± 0	2.87 ± 0.06	11.72 ± 1.03	2.44 ± 0.06	30.3 ± 1.8	ND	308.9 ± 61.1	52.3 ± 2.7	36.3 ± 0.8	66.7 ± 1.6	30.6 ± 1.7	46.6 ± 5.1	181.5 ± 4.2	65.4 ± 0.6	30.9 ± 0.6	5.20 ± 0.16	4.50 ± 0.56	28.6 ± 0.7
-NPK	1.6 ± 0.1	27 ± 0	1478 ± 13	204 ± 1	2.54 ± 0.01	6.62 ± 0.27	1.50 ± 0.03	17.1 ± 1.5	ND	516.5 ± 39.4	30.3 ± 0.4	43.6 ± 0.7	10.3 ± 0.1	26.0 ± 1.6	30.3 ± 0.4	87.8 ± 1.2	15.7 ± 0.2	16.2 ± 0.0	8.22 ± 0.09	5.71 ± 0.01	28.2 ± 0.3

Ammonium acetate extract

	P (μg g^{-1})	K (μg g^{-1})	Ca (μg g^{-1})	Mg (μg g^{-1})	Fe (μg g^{-1})	Mn (μg g^{-1})	Zn (μg g^{-1})	Cu (ng g^{-1})	Mo	B	Li (ng g^{-1})	Na (μg g^{-1})	Al (μg g^{-1})	Cr (ng g^{-1})	Co (ng g^{-1})	Ni (ng g^{-1})	As (ng g^{-1})	Cd (ng g^{-1})	Sr (μg g^{-1})	Ba (μg g^{-1})	Cs (μg g^{-1})
+NPK	14.2 ± 3.2	425 ± 18	3431 ± 138	169 ± 4	3.52 ± 0.20	11.52 ± 1.82	0.450 ± 0.088	5 ± 3	ND	ND	52.9 ± 5.8	42.5 ± 1.5	2.19 ± 0.09	169 ± 102	20.1 ± 2.2	124 ± 6	61.3 ± 1.9	90.6 ± 3.5	12.7 ± 0.2	28.6 ± 1.0	1.30 ± 0.06
-N	12.9 ± 2.0	747 ± 60	4026 ± 69	221 ± 3	3.63 ± 0.08	5.87 ± 0.11	0.375 ± 0.076	16 ± 16	ND	ND	48.0 ± 3.3	41.9 ± 4.0	0.70 ± 0.03	22 ± 22	13.3 ± 0.4	78 ± 2	69.5 ± 4.1	74.9 ± 3.1	20.0 ± 0.3	30.3 ± 0.9	0.93 ± 0.08
-P	3.9 ± 1.2	706 ± 23	2961 ± 92	215 ± 9	3.60 ± 0.46	16.95 ± 3.56	0.410 ± 0.059	116 ± 19	ND	ND	29.3 ± 5.9	38.4 ± 1.7	1.73 ± 0.38	304 ± 88	32.1 ± 6.9	89 ± 5	34.9 ± 2.7	56.7 ± 1.9	12.6 ± 0.3	34.5 ± 3.3	1.34 ± 0.03
-K	12.3 ± 0.7	114 ± 5	3719 ± 41	136 ± 2	4.58 ± 0.71	12.81 ± 1.73	0.340 ± 0.038	74 ± 23	ND	ND	51.9 ± 2.8	53.6 ± 2.8	2.99 ± 0.42	122 ± 73	25.4 ± 2.2	118 ± 9	66.7 ± 3.3	89.4 ± 0.8	14.3 ± 0.1	29.8 ± 0.7	3.77 ± 0.09
-NPK	6.3 ± 3.0	125 ± 8	3886 ± 12	435 ± 6	3.97 ± 0.47	6.74 ± 0.24	0.044 ± 0.031	82 ± 44	ND	ND	15.6 ± 1.2	63.1 ± 3.5	0.04 ± 0.01	109 ± 34	15.2 ± 0.9	60 ± 3	29.0 ± 2.1	44.4 ± 0.8	23.3 ± 0.2	37.5 ± 0.9	3.42 ± 0.06

ND: Not detected

Values are means (n = 3) ± standard errors.

Table S3 Concentration of each element in leaves of maize grown under different fetrtilizer treatment.

	N ($mg\ g^{-1}$)	P ($mg\ g^{-1}$)	K ($mg\ g^{-1}$)	Ca ($mg\ g^{-1}$)	Mg ($mg\ g^{-1}$)	Fe ($\mu g\ g^{-1}$)	Mn ($\mu g\ g^{-1}$)	Zn ($\mu g\ g^{-1}$)	Cu ($\mu g\ g^{-1}$)	Mo ($\mu g\ g^{-1}$)	B ($\mu g\ g^{-1}$)
+NPK	34.2 ± 0.4	3.81 ± 0.21	33.3 ± 1.6	4.38 ± 0.35	1.84 ± 0.15	118 ± 13	66.7 ± 7.7	34.4 ± 3.4	11.20 ± 0.81	0.19 ± 0.03	3.33 ± 0.07
-N	24.8 ± 0.5	2.78 ± 0.05	29.1 ± 0.7	3.30 ± 0.16	1.32 ± 0.03	102 ± 13	20.2 ± 1.3	20.8 ± 0.9	7.64 ± 0.52	1.27 ± 0.43	19.84 ± 6.47
-P	33.4 ± 1.2	1.43 ± 0.07	34.7 ± 0.9	3.64 ± 0.28	1.70 ± 0.09	148 ± 17	48.8 ± 1.3	37.6 ± 2.0	12.27 ± 0.09	0.31 ± 0.01	6.35 ± 0.62
-K	37.1 ± 0.7	6.24 ± 0.08	6.3 ± 0.1	8.20 ± 0.10	6.14 ± 0.09	232 ± 24	83.9 ± 1.8	54.6 ± 1.5	9.84 ± 0.55	0.34 ± 0.01	6.88 ± 0.33
-NPK	28.8 ± 0.7	2.42 ± 0.22	12.1 ± 2.7	4.15 ± 0.20	4.02 ± 0.44	205 ± 32	19.4 ± 1.8	33.5 ± 3.5	13.10 ± 1.39	1.40 ± 0.10	4.32 ± 0.14

Values are means (n = 3) ± standard errors.

Li ($\mu g\ g^{-1}$)	Na ($\mu g\ g^{-1}$)	Al ($\mu g\ g^{-1}$)	Cr ($\mu g\ g^{-1}$)	Co ($\mu g\ g^{-1}$)	Ni ($\mu g\ g^{-1}$)	As ($\mu g\ g^{-1}$)	Cd ($\mu g\ g^{-1}$)	Sr ($\mu g\ g^{-1}$)	Ba ($\mu g\ g^{-1}$)	Cs ($\mu g\ g^{-1}$)
0.066 ± 0.008	196 ± 6	39 ± 12	4.43 ± 0.87	0.05 ± 0.02	2.44 ± 0.44	0.155 ± 0.033	0.212 ± 0.019	11.9 ± 1.0	1.88 ± 0.15	2.99 ± 0.26
0.054 ± 0.032	194 ± 7	34 ± 7	5.49 ± 0.77	0.08 ± 0.01	2.83 ± 0.33	0.208 ± 0.057	0.139 ± 0.023	10.6 ± 0.6	1.91 ± 0.14	0.37 ± 0.04
0.055 ± 0.007	246 ± 17	84 ± 18	8.41 ± 1.51	0.14 ± 0.03	4.95 ± 1.02	0.174 ± 0.030	0.177 ± 0.011	10.1 ± 1.0	2.93 ± 0.43	0.56 ± 0.11
0.108 ± 0.015	339 ± 14	154 ± 14	11.29 ± 1.11	0.19 ± 0.03	6.29 ± 0.68	0.465 ± 0.028	0.272 ± 0.001	23.5 ± 0.4	4.89 ± 0.09	9.04 ± 0.12
0.127 ± 0.013	265 ± 28	139 ± 24	13.29 ± 4.10	0.19 ± 0.05	7.11 ± 1.94	0.276 ± 0.049	0.087 ± 0.005	18.4 ± 1.7	3.18 ± 0.32	8.20 ± 0.21

Table S4 Correlation coefficient between concentrations determined by the methods (i) and (ii).

(i)	(ii)	K	Ca	Mg	Fe	Mn	Zn	Cu	B	Mo	Li	Al	Na	Cr	Co	Ni	As	Cd	Sr	Ba	Cs	P
0.1 M HCl	1 M HCl	<0.99	<0.95	<0.90			<0.90					<0.90	<0.95	<0.80		<0.95	<0.90	<0.95	<0.99		x	<0.99
0.1 M HCl	Water	<0.95				<0.80			<0.80												x	<0.90
0.1 M HCl	Acetate	<0.99	<0.95	<0.99			<0.80		<0.80							<0.80			<0.95	<0.80	x	<0.95
0.1 M HCl	Ammonium acetate	<0.99	<0.90	<0.99												<0.90	<0.80	<0.90	<0.95		x	
0.1 M HCl	HNO_3 digestion	<0.95																<0.90	<0.95		x	<0.99
0.1 M HCl	Truog	x	x	x	x	x	x	x	x	x	x	x	x	x	x	x	x	x	x	x	x	<0.95
1 M HCl	0.1 M HCl	<0.99	<0.95	<0.90			<0.90					<0.90	<0.95	<0.80		<0.95	<0.90	<0.95	<0.99		x	<0.99
1 M HCl	Water	<0.90												<0.80			<0.80				x	<0.90
1 M HCl	Acetate	<0.99	<0.95	<0.90			<0.80									<0.80	<0.80	<0.80	<0.95		x	<0.95
1 M HCl	Ammonium acetate	<0.99	<0.95	<0.80					x	x						<0.80	<0.90	<0.90	<0.95		x	
1 M HCl	HNO_3 digestion	<0.95	<0.80					<0.80										<0.95	<0.95		x	<0.99
1 M HCl	Truog	x	x	x	x	x	x	x	x	x	x	x	x	x	x	x	x	x	x	x	x	<0.95
Water	0.1 M HCl	<0.95				<0.80			<0.80												x	<0.90
Water	1 M HCl	<0.90												<0.80			<0.80				x	<0.90
Water	Acetate	<0.90				<0.80			<0.80		<0.80	<0.90			<0.80		<0.95	<0.80			<0.80	<0.95
Water	Ammonium acetate	<0.90				<0.90			x	x					<0.80		<0.90				<0.80	
Water	HNO_3 digestion	<0.90																				<0.90
Water	Truog	x	x	x	x	x	x	x	x	x	x	x	x	x	x	x	x	x	x	x	x	<0.90
Acetate	0.1 M HCl	<0.99	<0.95	<0.99			<0.80		<0.80							<0.80			<0.95	<0.80	x	<0.95
Acetate	1 M HCl	<0.99	<0.95	<0.90			<0.80									<0.80	<0.80	<0.80	<0.95		x	<0.95
Acetate	Water	<0.90				<0.80			<0.80		<0.80	<0.90			<0.80		<0.95	<0.80			<0.80	<0.95
Acetate	Ammonium acetate	<0.99	<0.90	<0.99		<0.99	<0.90		x	x		<0.80	<0.90		<0.95	<0.90	<0.90	<0.95	<0.99		<0.99	
Acetate	HNO_3 digestion	<0.95	<0.80																<0.95			<0.95
Acetate	Truog	x	x	x	x	x	x	x	x	x	x	x	x	x	x	x	x	x	x	x	x	<0.95
Ammonium acetate	0.1 M HCl	<0.99	<0.90	<0.99												<0.90	<0.80	<0.90	<0.95		x	
Ammonium acetate	1 M HCl	<0.99	<0.95	<0.80					x	x						<0.80	<0.90	<0.90	<0.95		x	
Ammonium acetate	Water	<0.90				<0.90			x	x					<0.80		<0.90				<0.80	
Ammonium acetate	Acetate	<0.99	<0.90	<0.99		<0.99	<0.90		x	x		<0.80	<0.90		<0.95	<0.90	<0.90	<0.95	<0.99		<0.99	
Ammonium acetate	HNO_3 digestion	<0.95	<0.80						x	x								<0.80	<0.95			
Ammonium acetate	Truog	x	x	x	x	x	x	x	x	x	x	x	x	x	x	x	x	x	x	x	x	
HNO_3 digestion	0.1 M HCl	<0.95																<0.90	<0.95		x	<0.99
HNO_3 digestion	1 M HCl	<0.95	<0.80					<0.80										<0.95	<0.95		x	<0.99
HNO_3 digestion	Water	<0.90																				<0.90
HNO_3 digestion	Acetate	<0.95	<0.80																<0.95			<0.95
HNO_3 digestion	Ammonium acetate	<0.95	<0.80						x	x								<0.80	<0.95			
HNO_3 digestion	Truog	x	x	x	x	x	x	x	x	x	x	x	x	x	x	x	x	x	x	x	x	<0.99
Truog	0.1 M HCl	x	x	x	x	x	x	x	x	x	x	x	x	x	x	x	x	x	x	x	x	<0.95
Truog	1 M HCl	x	x	x	x	x	x	x	x	x	x	x	x	x	x	x	x	x	x	x	x	<0.95
Truog	Water	x	x	x	x	x	x	x	x	x	x	x	x	x	x	x	x	x	x	x	x	<0.90
Truog	Acetate	x	x	x	x	x	x	x	x	x	x	x	x	x	x	x	x	x	x	x	x	<0.95
Truog	Ammonium acetate	x	x	x	x	x	x	x	x	x	x	x	x	x	x	x	x	x	x	x	x	
Truog	HNO_3 digestion	x	x	x	x	x	x	x	x	x	x	x	x	x	x	x	x	x	x	x	x	<0.99

r: <0.99 / <0.95 / <0.90 / <0.80

X: Not determined or Not detected
